# Pot losses and associated implications in Barents sea snow crab fishery

**DOI:** 10.1038/s41598-025-99749-4

**Published:** 2025-04-29

**Authors:** Kristine Cerbule, Roger B. Larsen, Astrīda Rijkure

**Affiliations:** 1 School of Energy, Geoscience, Infrastructure and Society, Heriot-Watt University, Edinburgh, UK; 2 Riga Technical University, Riga, Latvia; 3 UiT The Arctic University of Norway, Tromsø, Norway; 4 University of Latvia, Riga, Latvia

**Keywords:** Snow crab, Abandoned, lost or otherwise discarded fishing gear, Ghost fishing, Environmental sciences, Environmental impact

## Abstract

Snow crab (*Chionoecetes opilio*) is considered an invasive species in the Barents Sea, with the first observations dating back to 1996. The Norwegian commercial snow crab fishery started in 2012. In this fishery conical baited pots are adopted, similar to fisheries in other areas that target snow crabs. Over the last decade, different management measures have been implemented to ensure sustainability in this relatively new fishery. One central challenge is pot loss during deployment caused by challenging weather and operational conditions. Lost snow crab pots exhibit considerable potential for the continuous capture of crabs, so-called ghost fishing, which has been documented during lost gear retrieval and experimental trials. This study accounted for different snow crab pot loss scenarios and associated economic implications. The results show that given the substantial number of pots on snow crab fishing vessels, even small variations in pot loss rates (pot losses ranging from 0.5 to 3.0%) could result in considerable differences in ghost fishing amounts and the associated environmental and economic effects. The estimated amounts of ghost fishing in this study ranged from 11.5 to nearly 70 tonnes of ghost-fished crabs over a 3-year period, assuming 0.5–3.0% pot loss scenarios, resulting in significant differences in the amount of ghost-fished snow crabs and the value of the ghost fishing catch. These results highlight the importance of incentives and technical measures that can reduce pot losses and the associated ghost fishing time.

## Introduction

Snow crab (*Chionoecetes opilio*) is a subarctic species that is found in different cold-water areas in the North Pacific, Bering Sea, Atlantic Ocean, and Arctic areas^1,2^. This species prefers environments with temperatures ranging from 0 to 5 °C and muddy or sandy seafloors down to depths of 450 m^2,3^. Snow crab is fished in many regions and is considered a commercial species, with catches varying across different regions, including waters off Canada, Alaska, Greenland and Russia.

In the Barents Sea region, the snow crab is considered an invasive species, with the first observations dating back to 1996^4^. Earlier studies revealed that snow crabs migrated to these regions^5^, followed by their subsequent spread. The Norwegian fishery for snow crabs in the Barents Sea has developed since 2012, and the first commercial catches reached only 2.5 tonnes^6^. Since then, this fishery has grown rapidly, and the total catch obtained using conical pots during the fishing season from January to March in 2024 reached 10,300 tonnes, i.e., an increase of 30% from 2022 due to an increase in the quota^7^. The quota for 2025 increased by 30% to 12,750 tonnes^8^. It has been estimated that the conditions for snow crabs in the Barents Sea are optimal in terms of both environmental conditions and food availability^9,10^. Therefore, it is projected that this fishery will increase and that snow crabs will spread further north and west towards the Svalbard Islands due to the increasing temperatures and food availability^11^.

Since the beginning of the snow crab fishery in the Barents Sea, several regulations have been implemented to address different fishery sustainability issues and ensure that the stock is not depleted. Specifically, a closed season for snow crabs was implemented in 2017 to avoid the capture of soft-shelled crabs and to protect snow crabs during and after moulting. Currently, the closed season ranges from the 1st of July to the 30th of November each year. Total allowable catches (TACs) have been implemented since 2017 on the basis of annual surveys aiming to map snow crab densities. Furthermore, a minimum landing size (MLS) for snow crabs was implemented, which was initially set to a carapace width (CW) of 100 mm^12^. This CW value decreased to 95 mm in 2020^5^ and now matches the MLS value in the Canadian snow crab fishery^13^, whereas a value of 100 mm is used in the snow crab fishery in Greenland^14^. Thus, the Norwegian fishery targets large male crabs since female snow crabs do not reach an MLS value of 95 mm CW^2^.

Size selection in crab pots depends on the mesh size (130–140 mm), which allows undersized individuals to escape during gear deployment on the seabed. Furthermore, all undersized snow crabs that are captured must be released back to the sea. However, the released crabs have an unknown survival rate. In 2020, the maximum number of pots allowed on each vessel was set to 9,000^15^. From the beginning of the fishing season in January 2025, the total number was reduced to 8,000 pots^16^. Over the years, a varying number of vessels have received licences to catch snow crabs in the Barents Sea (the total number of vessels in 2023 reached 69); however, only 20–40% of these vessels actively participated in fishing activities during the 2020–2024 period^17,18^. Fishing in the Barents Sea usually occurs from the beginning of January until the TAC is reached. No individual catch quotas are allocated to vessels, and active fishing vessels aim to maximize their catches (deploying pot lines with up to 440 pots on a single line) and engage in long trips lasting approximately 4–6 weeks. Thus, the TAC is reached by early spring, with the fishing season lasting a few months (e.g., in 2024, the TAC was reached by the 19th of March). Since 2025, quotas have been allocated to vessels that have actively participated in snow crab fishing to limit excessive fishing^19^.

One of the most significant sustainability challenges in the snow crab fishery is related to abandoned, lost, or otherwise discarded fishing gear (ALDFG). Specifically, pots that are left at sea, either intentionally or unintentionally, exhibit the potential to continue capturing crabs and cause mortality (so-called ghost fishing), resulting in marine pollution. Snow crab fishing in the Barents Sea is conducted in areas far offshore, with associated demanding weather and ice conditions that can result in pot losses and pot retrieval challenges. The total loss rates of pots in the snow crab fishery in the Barents Sea have not yet been quantified but are considered substantial according to reports after the closing of the snow crab fishing season from shrimp-trawl skippers operating in the same area who catch such pots in their nets^20,21^.

**Fig. 1:**
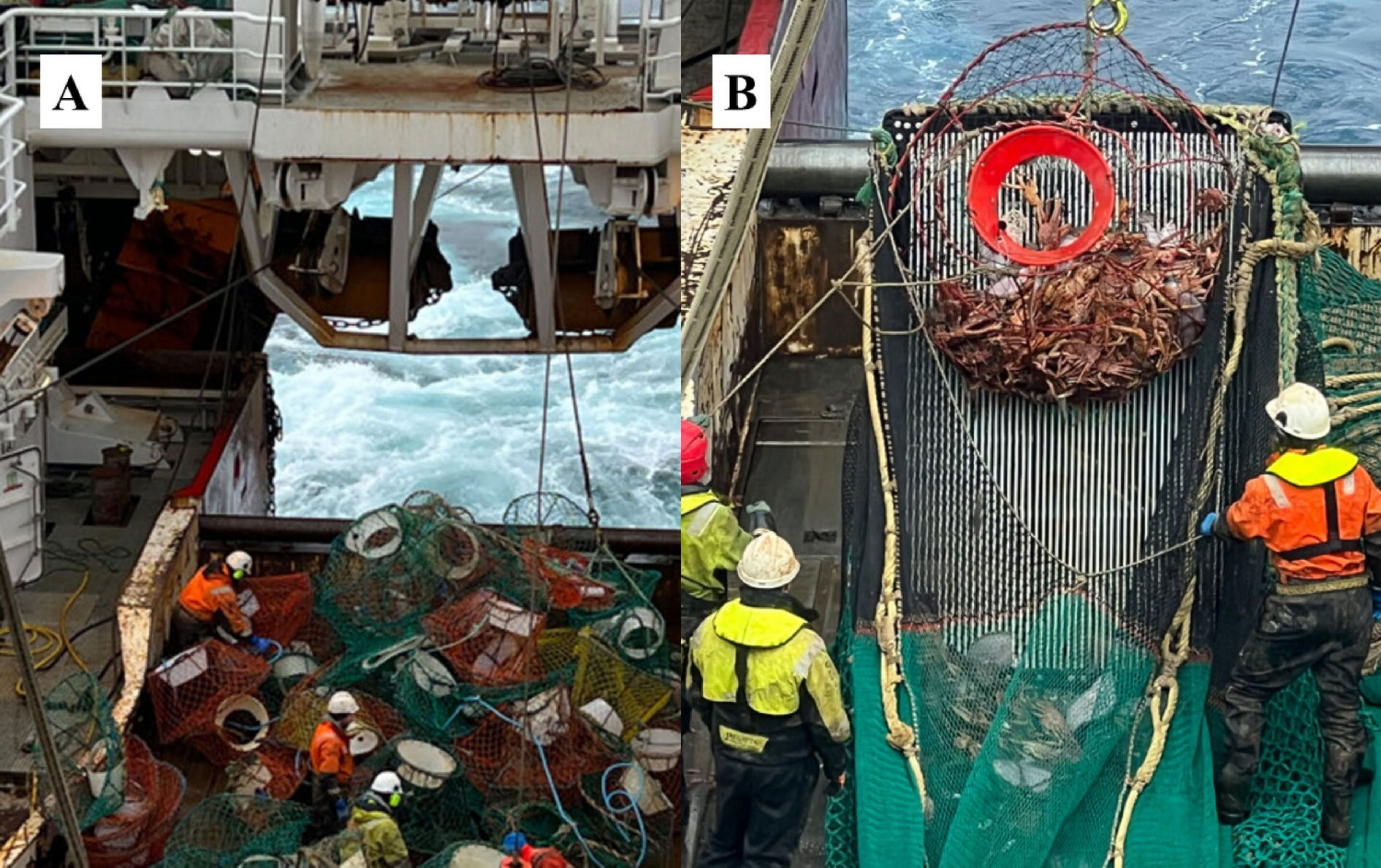
Images showing the implications of lost gear in the snow crab pot fishery in the Barents Sea. Image 1 A: Lost snow crab pots entangled in shrimp trawl fishing gear in the same area. Image 1B: Entangled lost pot containing ghost-fished snow crabs. Image source: Eirik A. Remøy (published by Engø^23^).

Following the results of annual lost gear retrieval operations in Norway, several thousand abandoned, lost or discarded (ALD) pots have been recovered^22^, including lost snow crab pots. However, gear retrieval in these areas far from the coast is challenging, time-consuming and expensive. Therefore, many pots lost over the years since fishery commencement may remain on the seabed, as the results of gear retrieval operations may cover only a limited share of the total lost gear. Furthermore, lost gear can accidentally become entangled with other pot lines, such as shrimp trawls in this fishing area, causing gear collisions (see, for example, Fig. 1a).

The incidental results of retrieved lost snow crab pots include catches of snow crabs (Fig. 1b)^23^. These results have also been documented in earlier scientific studies, with 43% of recovered lost snow crab pots containing ghost-fished snow crabs^24^. This confirms that in addition to marine pollution, lost gear can continue to capture snow crabs for prolonged periods. During experimental trials aiming to estimate the extent of ghost fishing in snow crab fishery, the results revealed that there was a significant amount of potential capture and mortality by ALD pots. Ghost fishing was estimated to constitute up to 8.3% on average relative to catches of commercial baited pots (confidence interval (CI): 4.3–13.7%)^25^. Variations in ghost fishing in snow crab pots have also been observed in other studies focused on the Canadian snow crab fishery^26,27^.

In 2024, the Norwegian Fisheries Directorate introduced the mandatory use of biodegradable twine in snow crab pots to reduce the ghost fishing time when pots are lost at sea. This twine should be made of cotton (noncoated and without a core) with a maximum twine diameter of 5 mm. Biodegradable twine should be incorporated into pot netting such that twine breakage would create an opening of at least 20 cm in diameter^28^. Biodegradable twine is used in pot fisheries elsewhere and is mandatory in the Canadian snow crab fishery^29^. Biodegradable (cotton) twine degrades much faster than ordinary plastic (polyethylene) netting does in pots and creates an opening in the pot netting, allowing animals that enter the gear to subsequently escape. However, during the period between 2012 and 2024, no such mechanism was used in the snow crab fishery. Therefore, pots lost at sea during this 12-year period could continue ghost fishing for several years since they are made of fully nonbiodegradable materials (a metal frame covered with polyethylene netting). Therefore, the introduction of cotton twine into pot construction is a promising development. However, one should consider both already lost and unrecovered gear and that lost pots with cotton twine included could provide ghost fishing for a period until twine degradation completion. This period can vary depending on the twine thickness and type used and the environmental conditions.

Continuous ghost fishing by lost pots can negatively impact the snow crab stock as unintended fishing mortality (bycatches of other species in this fishery are limited). In this way, ghost fishing by ALD pots in the snow crab fishery can restrict both the sustainability and profitability of the industry. Earlier observations and studies have initially focused on estimating the extent of ghost fishing by such lost pots after bait odour disappearance^24^, the potential extent of continuously captured snow crabs, and whether the presence of dead snow crabs could increase ghost fishing rates^30^. Such information, combined with estimates of potential gear losses, would allow the estimation of the negative economic effects of gear losses in this fishery.

Therefore, this study aimed to answer the following questions:

1. What is the potential extent of snow crab ghost fishing over time considering different scenarios of pot loss rates?
2. What is the value of the snow crab loss due to ghost fishing under each scenario?
3. Can changes in pot loss rates between scenarios result in significant differences in the estimated amount of ghost fishing and the lost value caused by ghost fishing?

## Materials and methods

### Acquiring data and estimates for the commercial snow crab fishery

This study focused on quantifying the long-term effects of lost pots expressed as the amount of ghost-fished snow crabs and the associated economic losses caused by ghost fishing, including managing the uncertainties associated with these estimates. Data were collected from a variety of sources, including direct field observations of the extent of ghost fishing, records from fishery and management institutions, and information from earlier scientific studies. These data include (1) the number of pots deployed, (2) commercial catch rates during one fishing season, (3) the estimated potential number of pots lost during one fishing season, and (4) the associated ghost fishing rates. Therefore, we implemented a series of methodological steps that allowed us to estimate the impacts and economic implications of ghost fishing. Each step of the process was designed to address specific aspects of the problem.

The first step of the analysis was to define the primary parameters related to the ghost fishing and snow crab catch data. In total, 20 vessels participated in the Norwegian snow crab fishery, and their total landings ranged from 50 to 1,230 tonnes in 2024^31^. The 10 best vessels contributed 75% to the total landed snow crabs with, to our knowledge, the maximum number of allowed pots. During the 2022–2024 seasons, the same number of snow crab vessels caught the majority of the Norwegian snow crab quota. The total number of pots used annually, denoted as *P*_*total*​_, was accordingly set to 90,000 in our estimations. This figure represents the estimated total amount of fishing gear deployed over one fishing season in the Norwegian snow crab fishery.

The total snow crab catch, *C*, from January to March in 2024 reached 10,300 tonnes, indicating that crabs are captured during approximately 3–4 months of the fishing season^32^. The price of each kg of frozen snow crab clusters consisting of snow crab legs was set to NOK 180, reflecting the market price of snow crabs in 2024^33^.

The catch rate in the commercial fishery was observed over a four-month period, corresponding to the active fishing season until the TAC for that year was reached^31^. To estimate the catch rate during the period when pots are lost and exposed to ghost fishing, we adjusted the observed catch rate to account for the prolonged period over which ghost fishing persists. In this study, we proposed the adjusted ghost fishing catch rate per pot (*CR*_*adjusted*_), which can be calculated as follows:

1 $$\:{CR}_{djusted}=\frac{C/4}{{P}_{deployed}}\times\:CF$$

where *P*_*deployed*_ is the monthly number of pots deployed during the fishing season, which lasts four months^17^. Furthermore, in Eq. (1), *CF* denotes a conversion factor of 1.61^33^, which accounts for the difference in the weight of landed snow crab leg clusters from that of live target-sized individuals (CW ≥ 95 mm). Accounting for this conversion factor allows us to estimate the quantity of ghost-fished crabs relative to the catches of commercial snow crab pots, where the weight of landed individuals is expressed as frozen clusters. Specifically, via the use of the conversion factor, the measurement of crabs caught is adjusted and expressed as the weight of the clusters to the total amount of whole crabs. This step is crucial, as it allows the analysis and comparison of catch rates in a consistent manner during the subsequent assessments of ghost-fished catches.

### Estimating the number of abandoned, lost, or discarded pots

Understanding the extent of lost pots during one fishing season is necessary to evaluate how many pots contribute to ghost fishing. The exact total number of lost pots in this fishery during each fishing season is unknown^17,29^. Therefore, in this study, we assumed four scenarios of varying pot loss rates on the basis of estimates reported in the literature regarding other pot fisheries and average gear loss rates^34^, and we estimated overall fishing gear losses. Studies in the Canadian snow crab fishery have shown that pot losses can cause an annual mortality of more than 600 tonnes of crabs, which corresponds to an approximately 2% annual pot loss^29,35^. Therefore, we applied a pragmatic approach and considered four scenarios in which the pot loss rate equalled 0.5%, 1%, 2%, and 3% of all individual pot deployments within the season. To estimate the impact of ghost fishing, we first calculated the number of lost pots as follows:

2 $$\:{P}_{lost}=\:{P}_{deployed}\times\:L$$

where *L* is the assumed pot loss rate (*L* = {0.005, 0.01, 0.02, 0.03}).

### Estimating the extent of ghost fishing by abandoned, lost or discarded pots

The time span for analysis in this study was set to 36 months. This provided a temporal context for the analysis. Specifically, given that pots lost during one season continue capturing crabs for three consecutive years, we estimated the development of ghost fishing over this period and the economic losses associated with ghost-fished catches^25^. An earlier study by Humborstad et al. (2021) indicated that ALD snow crab pots can continue to ghost fish for 1.5 years in the marine environment despite the presence of biodegradable twine^24^. The use of biodegradable components was not mandatory in the Norwegian snow crab pot fishery until 2024. Therefore, it can be assumed that such ALD pots can continue ghost fishing past this period. Therefore, in this study, a three-year period was set to obtain results over a longer period of ghost fishing, thereby avoiding extrapolation over longer periods.

**Fig. 2:**
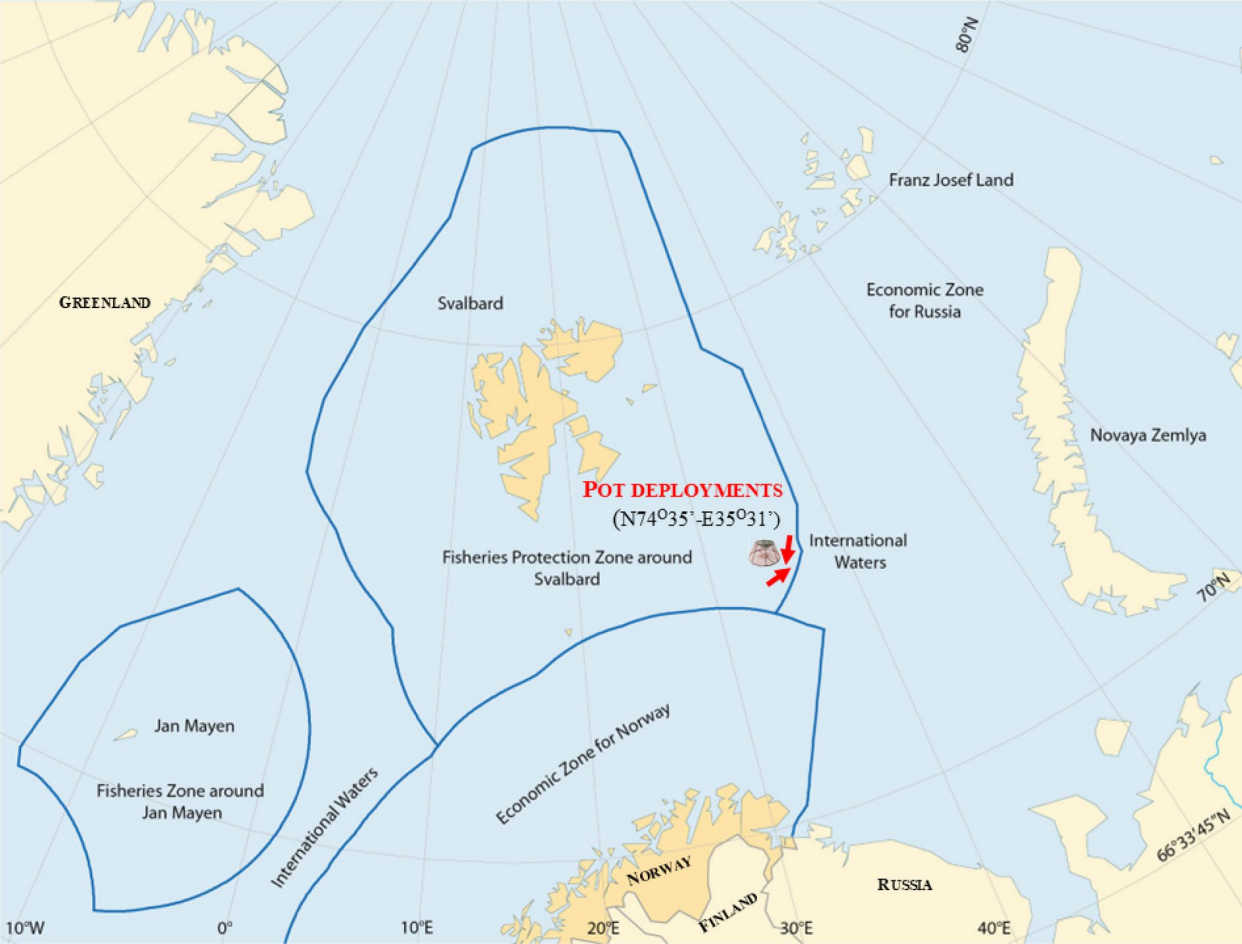
Map of the area where snow crab fishing occurs, with the arrows denoting the areas where data were collected to estimate the initial ghost fishing rate^25^ and the ghost fishing rate in the presence of dead conspecifics^30^.

In this study, the impact of ghost fishing was expressed as the lost pot ghost fishing efficiency relative to the catch efficiency of commercial pots. To quantify this metric for the snow crab fishery, the impact of ALD pots over time was calculated^25,30^. The estimated initial average ghost fishing efficiency of experimental ghost fishing snow crab pots (without bait) in the Barents Sea fishery was estimated as 8.3% (CI: 4.3–13.7%)), which is greater than the catch efficiency of commercial baited snow crab pots for target-sized crabs over a CW of 95 mm^25^. However, a decrease in ghost fishing could occur due to snow crab mortality, which could potentially repel other crabs from approaching^36,37^. The observed ghost fishing rate was 0.4% lower (CI: 0.2–0.6%) than that of commercial (baited) snow crab pots^30^ in the same area (Fig. 2). Notably, with the decomposition of dead individuals, the empty pots would increasingly demonstrate an increase in the extent of ghost fishing and continue capturing new crabs. This could, therefore, constitute several cycles of ghost fishing when pots are lost at sea and continue to capture snow crabs until other mechanisms (i.e., biodegradable twine^29^) eventually cause the cessation of ghost fishing.

We adjusted the monthly catch rate to account for the presence of ghost fishing pots in each scenario. Specifically, to represent variations in ghost fishing rates over time, we defined two levels of the ghost fishing efficiency: an initial higher efficiency (*E*_*initial*_) and a subsequent lower efficiency due to the presence of dead crabs (*E*_*low*_). We assumed a scenario in which snow crabs were captured at the initial rate for the first 6 months after pots were lost at sea. Earlier experiments have shown that crabs can survive without food sources for prolonged periods^38–40^. Specifically, studies have revealed that snow crabs can survive without feeding for 100 days, with a low risk of physical injuries^40^, but the lipid content is affected after 5 months of starvation^39^. Therefore, we assumed that the phase of initial ghost fishing would continue for a period of six months, i.e., until the initial expected mortality of ghost-fished snow crabs was reached. Thereafter, we assumed that the ghost fishing rate would decrease for a period of one month, during which the disintegration and decay of dead snow crab carapaces occurred. This was followed by a gradual increase in the ghost fishing rate up to the initial level of 8.3%^25^. The efficiency in each month (*E*_*month*_) was determined as follows:

3 $$\:{E}_{month}=\left\{\begin{array}{c}{E}_{initial}\:if\:1\:\le\:\:m\:\le\:6\\\:{E}_{low}\:if\:m=7\\\:{E}_{low}+\:\frac{{E}_{initial}-{E}_{low}}{5}\:\times\:\left(m-7\right)\:if\:8\:\le\:\:\le\:12\end{array}\right.$$

where *m* is the month. We applied this equation to estimate the ghost fishing efficiency during the remaining two years of the study period.

This equation captures the catch behaviour of ghost fishing pots: initially, these pots can effectively catch crabs in a continuous manner (rates according to^25^), but over time, their ghost fishing efficiency decreases due to mortality^30^) before gradually regaining efficiency. This approach captures the dynamic nature of ghost fishing and is essential for accurately estimating the impact of ghost fishing over the entire study period. The ghost fishing efficiency of lost pots was calculated with the initial efficiency and a lower efficiency value, both of which were obtained considering confidence intervals based on both estimates^25,30^.

We then adjusted the efficiency in each month to account for the presence of ghost fishing pots on the basis of the number of crabs caught by one ghost fishing pot (*g*) during a specific month *t*, considering a specific pot loss scenario *j* (ranging from 0.5 to 3.0%). This value was calculated as follows:

4 $$\:{g}_{t,\:\:j}=\:{P}_{lost}\:\times\:{E}_{month}\:\times\:\:{CR}_{adjusted}$$

The cumulative ghost fishing amount over the entire period for each scenario *G*_*j*_ was computed by summing the monthly (*m*) ghost catches for each scenario separately:

5 $$\:{G}_{j}=\:\sum\:_{t=1}^{m}{g}_{t,j}$$

### Estimating the economic loss caused by ghost fishing in each scenario

Within the context of ghost fishing, the economic value refers to the potential income of the industry that is lost because crabs are caught in ghost fishing pots and subsequently die without being harvested and sold. Moreover, the impacts of ghost fishing can be considerable if expressed as a fraction of the harvest in the commercial fishery. The estimation of the direct economic value in this study is based on several factors, including the weight of ghost-fished crabs, the value of frozen crab clusters, and the duration over which these losses occur.

The total extent of ghost fishing in each scenario was then used to estimate the economic value of the ghost-fished catch. This was conducted in each scenario separately by accounting for the individual ghost-fished catches in each scenario, which were multiplied by the estimated catch value (expressed as the value of frozen crab clusters obtained via the conversion factor *CF*).

### Accounting for uncertainties in estimations

Given the uncertainties in the ghost fishing efficiency and the other parameters involved, a bootstrapping technique was employed to estimate economic losses. This involved generating numerous scenarios (*i* = 1000) to account for variability in the estimations. Specifically, for ghost fishing estimations, different efficiencies were obtained during each iteration to reflect the uncertainty in how lost pots effectively continue to catch crabs over time within the limits set in previous field experiments of initial ghost fishing without bait presence^25^ and of pots containing dead snow crabs^2^ (simulated self-baiting). Thus, during each iteration *i*, the following steps were repeated:

1. Efficiency sampling: Ghost fishing efficiency values were sampled with replacement from their respective distributions.
2. Crab catch calculation: The number of crabs caught by ghost fishing was calculated for each month over the 36-month period via Eq. (3).
3. Economic value calculation: The monthly economic value of ghost-fished snow crabs was calculated, and the values were summed to obtain the total value for that iteration.

Finally, the results from all the iterations were aggregated to provide the mean value of the ghost-fished snow crab catch for each of the four scenarios, which represents the expected economic impact of ghost fishing. Furthermore, confidence intervals, in which the 2.5th and 97.5th quantiles of the economic losses are calculated to provide 95% CIs, offer insight into the range of the potential impacts of ghost fishing. The resulting mean estimates for ghost fishing and the associated lost value for individual scenarios were reported as the best estimates with associated uncertainties.

## Results

### Estimated pot loss and ghost fishing rate

**Fig. 3:**
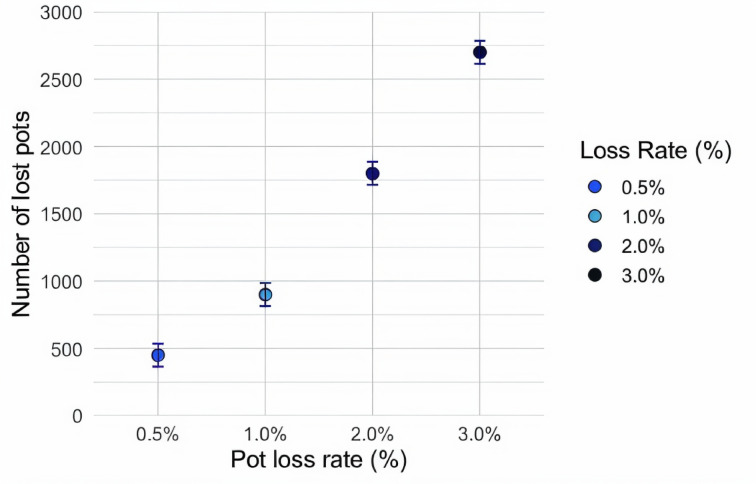
Number of snow crab pots lost during one fishing season under the different scenarios of pot loss rates considering pot deployment during the fishing season (90,000 pots deployed and estimated loss rates of 0.5%, 1.0%, 2.0% and 3.0%).

The estimated loss of pots over one season varied considerably among the different scenarios (Fig. 3). Specifically, considering that 10 vessels employed the maximum number of pots over one fishing season, the pot loss rates over one fishing season ranged from 450 (CI: 365–536) to 2,699 (CI: 2,615–2,785) pots, which can contribute to ghost fishing.

**Fig. 4:**
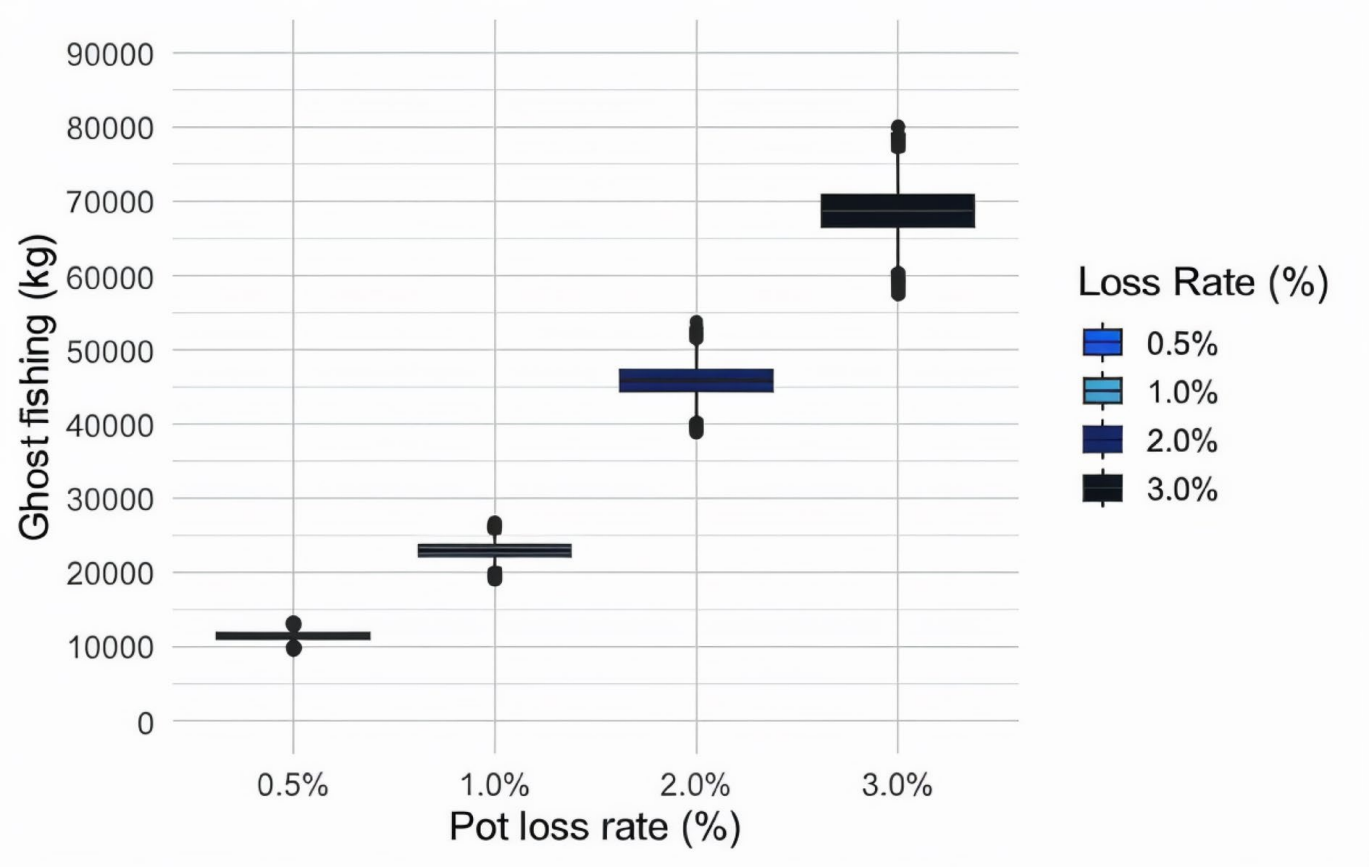
Ghost-fished catch (kg) over a 36-month period following pot loss occurrence under each pot loss scenario.

The pot loss rates directly affected the potential ghost fishing catches over the 36-month period after pot loss occurrence. During this period, we assumed different ghost fishing rates due to the presence/absence of dead snow crabs^25,30^, thus causing variations in ghost fishing over time. This trend was similar across all the scenarios, but due to the different amounts of assumed lost pots, the resulting ghost fishing rates differed between the scenarios (pot losses ranging from 0.5 to 3%) throughout this period (Fig. 4).

**Fig. 5:**
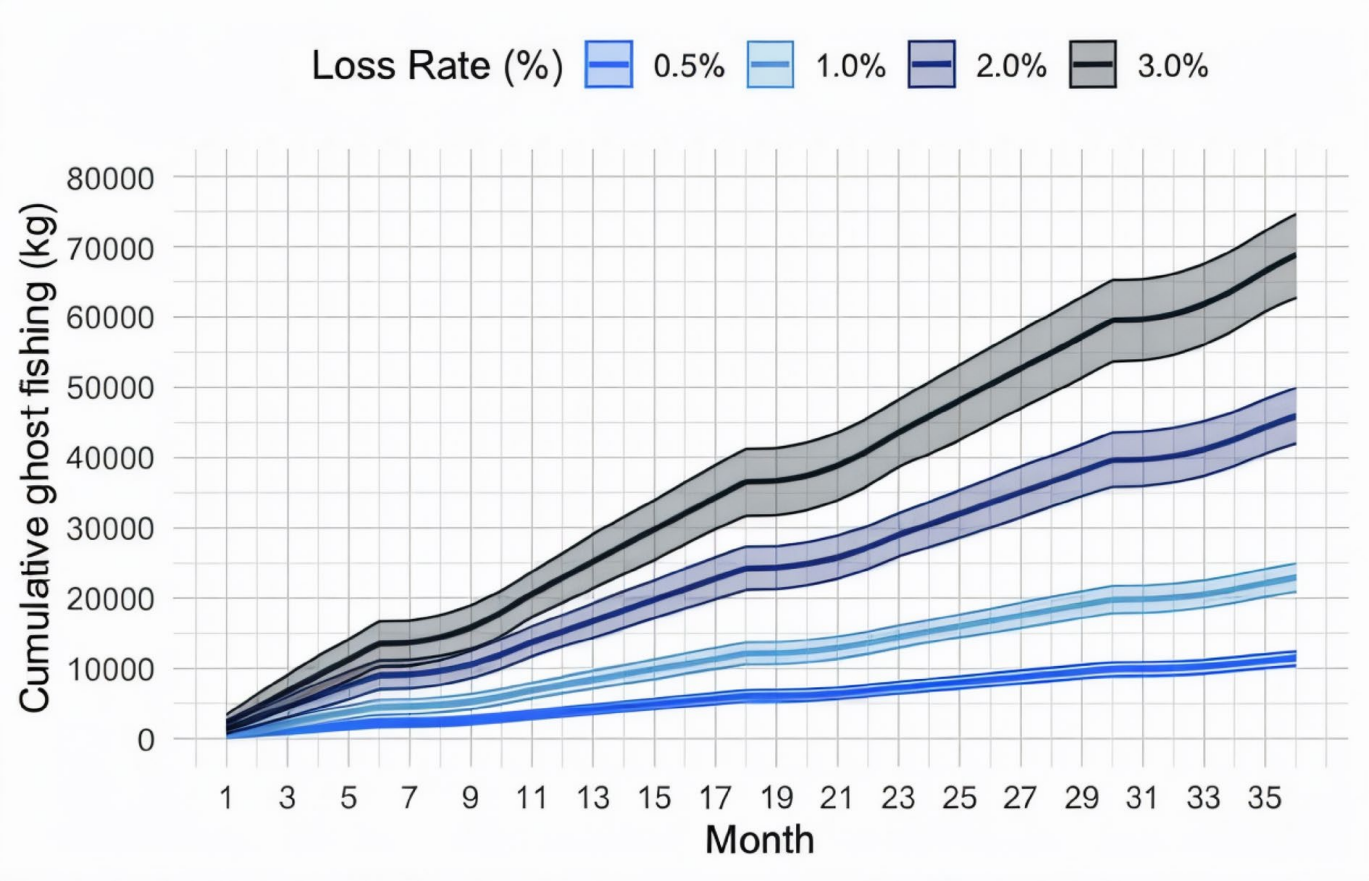
Cumulative estimated amounts (kg) of ghost-fished snow crabs under each scenario over a period of 36 months.

Even when adjusting the ghost fishing rate over time due to the presence or absence of dead conspecifics in the pots, the expected ghost-fished amount would increase over the period considered, yielding increasingly high ghost fishing rates over time. Therefore, the cumulative number of ghost-fished crabs continued to increase, and this increase significantly differed among the four scenarios considered because of the amount of assumed lost gear. The cumulative amount of ghost-fished snow crabs by weight (expressed as whole crabs) is shown in Fig. 5.

### Economic value of ghost-fished snow crabs in the Barents sea

The summed ghost-fished catch over the 36-month period reached approximately 11.5 tonnes of whole crabs (11.434 tonnes; 95% CI 10.438–12.434 tonnes) under the 0.5% pot loss scenario to almost 70 tonnes of whole crabs (68.901 tonnes, 95% CI 62.710–74.945 tonnes) under the 3% pot loss scenario (Table 1).

If these crabs were captured commercially, the corresponding value (frozen crab clusters) would vary between 106,751 NOK (CI: 97,023–116,146 NOK) and 641,342 NOK (CI: 585,022–698,537 NOK) (which equals 9,126–54,829 EUR) (Table 1).

**Table 1:** Total ghost-fished snow crab over 36 months for each scenario with estimated ghost fished weights and economical value of ghost fished catches.

Scenario	Percentage of lost pots of pots deployed	Ghost fishing extent over 36 months (tons whole crab)	Estimated lost value over 36 months (NOK)
1	0.5%	11.434 (10.438–12.434)	1 282 665 (1 172 004 − 1 389 905)
2	1.0%	22.890 (20.757–24.906)	2 559 758 (2 341 244-2 777 560)
3	2.0%	45.945 (41.825–49.598)	5 127 417 (4 671 769-5 581 927)
4	3.0%	68.901 (62.710-74.945)	7 692 010 (7 031 133-8 336 982)

Values in parentheses are 95% confidence intervals.

**Fig. 6:**
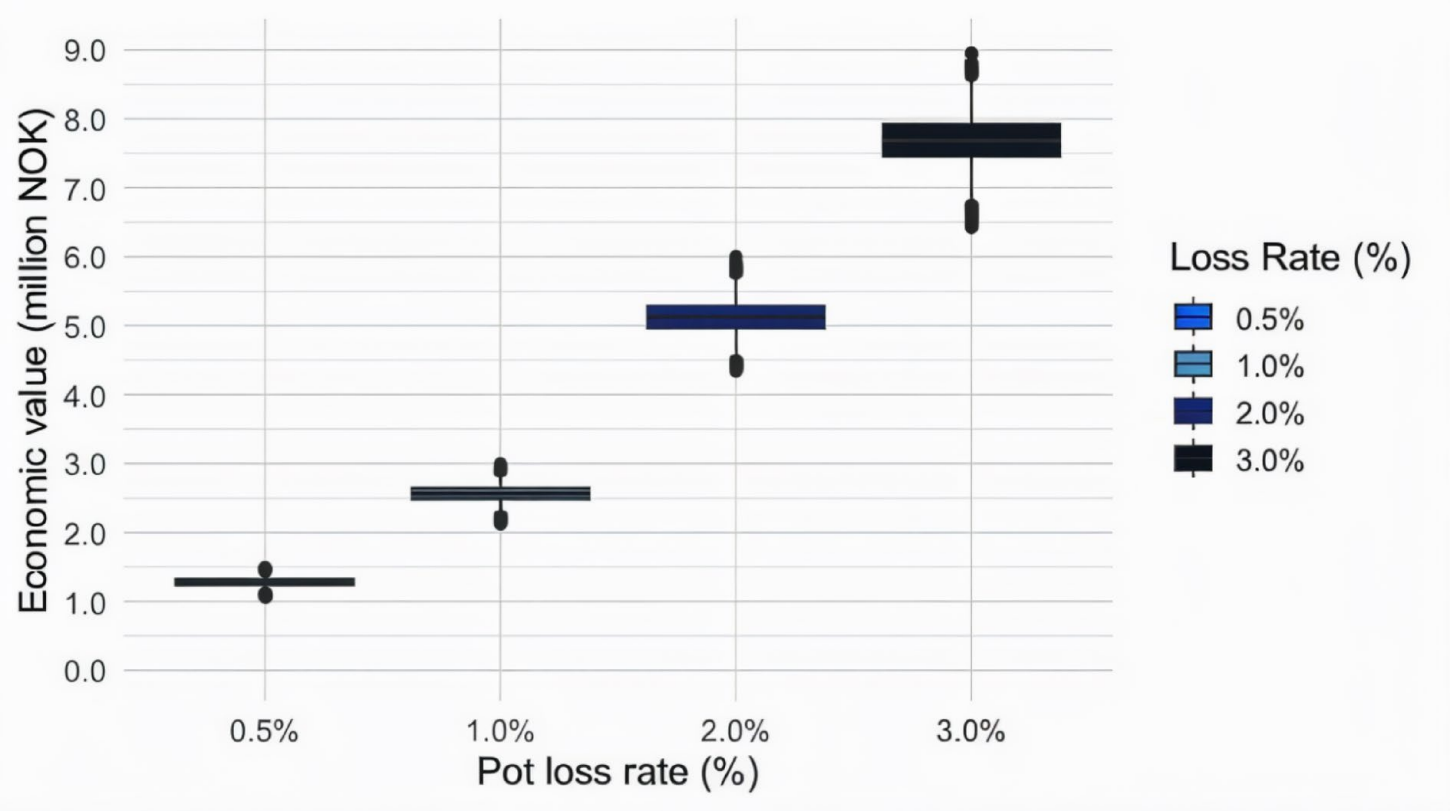
Estimated economic value (in NOK) lost due to ghost-fished snow crabs under each scenario over a period of 36 months.

The distributions of the direct economic value lost due to ghost fishing under the scenarios with varying pot loss rates are shown in Fig. 6. These losses highlight only the direct lost catch without reflecting other economic implications of ghost fishing.

The observed differences in economic values between the various scenarios were considerable, especially when comparing the 0.5% and 3% pot loss scenarios. This finding shows that relatively small differences in pot loss rates can cause considerable differences in the implications for the fishery (Fig. 6).

## Discussion

This study aimed to summarize the current knowledge and estimate the extent of ghost fishing in the snow crab fishery in the Barents Sea by ALD pots. The precise number of gear lost at sea during each fishing season in this fishery is unknown, even though fishermen acknowledge that the number of lost pots can be significant^17^. It is estimated that large numbers of gear can accumulate on the seafloor since the fishery has been initiated in this area^24^. This reflects results from other snow crab fisheries, such as that in Canada, in which the same pot design is used as that deployed in the Barents Sea^29,35^.

Snow crab fishing in the Barents Sea is divided between Russia and Norway. The total allowable catches (TACs) are set at the national level, and Russia attained TACs of 13,912 and 22,475 tonnes in 2023 and 2024, respectively, according to the Federal Agency for Fishery of the Russian Government. This value is approximately two times greater than the Norwegian TAC^41^. We have very little insight into the fishing patterns of Russian vessels, other than the use of conical pots. Vessels from Russia have no access to snow crab grounds in the Norwegian zone, and Norwegian vessels cannot fish in the Russian zone. Russian snow crab fishing occurs in the eastern part of the Barents Sea (near Novaya Zemlya, as shown in Fig. 2) and is not considered to affect Norwegian captures.

Fishing gear loss rates can vary considerably due to factors such as the fishing area (seabed type, currents, depth, weather conditions, etc.), gear type, and potential gear conflicts^35,42–44^]. The average estimated fishing gear loss rate globally reaches 2% of all the fishing gear used^34^. ALD pots are usually perceived as gear with a substantial risk of negative ecological impacts due to ghost fishing^43^. For snow crab fishing specifically, earlier studies in Canada have revealed that approximately 2% of all snow crab pots used during a given season are lost^29,35^. Considering that many pots are deployed, this can contribute considerably to ghost fishing. Similar estimates of the pot loss in the Barents Sea are not available. However, lost gear is accidently captured by shrimp trawlers operating in the same fishing area following the snow crab fishing season^17,23^ and is retrieved during clean-up operations organized by the Norwegian Directorate of Fisheries^22^.

This study aimed to provide an overview of what happens with gear lost over one fishing season. One must consider that more pots are lost each year, so this would not reflect the total ghost fishing implications but just a fraction of the ghost gear lost during one season.

There are also other ecological consequences of ghost fishing (e.g., reduced recruitment) that should be considered. The mesh size used in snow crab pots allows the escape of smaller crabs (essentially those under an MLS of a 95-mm CW) while retaining larger male crabs over the MLS. Therefore, in the case of pot loss, larger snow crabs would be exposed to ghost fishing risk since they would be unable to escape through the mesh openings (i.e., 140-mm mesh size). Loss of these larger crabs reduces recruitment, which can negatively affect the snow crab stock size, as large male snow crabs are important for preserving the reproductive capacity of snow crab populations^45^. The Norwegian snow crab stock is currently estimated to be healthy, and fishing efforts and TACs have been increasing yearly since 2017^17,41^. Therefore, we cannot assume that ghost fishing has thus far affected the stock in a way that would reduce its size and result in a decline in the commercial fishery. However, the increasing number of gear lost over the years and the associated extent of ghost fishing have yielded negative environmental effects and have led to questions regarding the sustainability of this fishery.

Since the beginning of 2024, the use of biodegradable cotton twine in pots has been mandatory in the Barents Sea snow crab fishery^28^. This can considerably reduce the potential ghost fishing time^29^. These mechanisms are similar to those used in the Canadian snow crab fishery. An earlier study in Canadian waters revealed that the time for cotton twine degradation and the subsequent release of snow crabs of all sizes is approximately 1.5 years from exposing the gear to the marine environment^29^. The degradation time depends on the type of twine, the environmental conditions, and how long the twine has been used prior to pot loss occurrence^46^. Notably, the reduction in the ghost fishing time can be considerable, e.g., at least half our 36-month estimated amount of ghost fishing.

The results of this study must be interpreted with caution, as the estimates are based on limited information on fishery activities and current scientific results, such as, for example, unknown and assumed pot loss rates and ghost fishing durations. In several pot fisheries, including the snow crab fishery, potential self-baiting is often considered to potentially increase ghost fishing rates^24,27^. However, avoiding dead conspecifics is associated with behaviour related to avoiding potential danger, which has been observed in several other crab species^37,38^. In this study, we used existing estimates of snow crab ghost fishing in the Barents Sea fishery while considering these aspects; therefore, it is based on available scientific information on ghost fishing. In actual ALDFG scenarios, other factors could affect ghost fishing rates. For example, future studies should aim to determine whether live conspecifics in gear could affect ghost fishing rates in ALD pots. Furthermore, the presence of other organisms can impact ghost fishing rates; however, the bycatch rates in the snow crab fishery are generally very low^47–49^.

On the basis of our estimates, the results showed that even small changes in pot loss rates can result in significant differences in ghost fishing. Therefore, efforts should first be made to prevent the occurrence of ALD pots. The snow crab fishery in Norway is regulated according to an open-access and Olympic fishery model, suggesting that each vessel will use a maximum effort to capture as much as possible until the total crab quota is reached. Enforcement of vessel quotas are being examined as a harvesting rule to prevent pot losses^17^. Regulations limiting the number of boats in the Norwegian snow crab fishery have been set since 2025^31^. Another way to reduce the amount of ALDFG is gear marking with a visible highflyer, flag, light signal, radar reflectors and buoys with enough capacity to maintain the highflyer (and buoys) at the surface regardless of the drag forces on the buoy lines. Furthermore, technical solutions, e.g., radio frequency identification tags to increase the chances of locating and retrieving gear lost due to drift ice or gear conflicts, should be further developed^50^.

Importantly, such technical measures (i.e., gear marking and radio frequency identification tags) can limit the gear loss but not prevent all pot losses in this fishery, and not all pots lost at sea can be subsequently retrieved because of the large depths and challenging weather conditions, among other factors. Therefore, additional measures for reducing the ghost fishing time, such as the use of cotton twine for providing an escape mechanism, are crucial in similar pot fisheries, such as in the Canadian and Norwegian snow crab fisheries. Such mechanisms can be improved by the use of other biodegradable elements in pot construction, such as biodegradable escape panels (also called escape vents or escape gaps in the literature), which degrade faster over time if pots are lost and left in the ocean. This would then disable ALD pots by allowing the escape of snow crabs of all sizes and incidental bycatches of other species. Furthermore, polyethylene (PE) net panels (pot-jackets) or sections of PE mesh panels can be replaced with biodegradable nets^51^.

## Conclusion

This study focused on ghost fishing and the associated value of lost ghost-fished catches captured in pots lost during a single fishing season, thereby providing a snapshot of the impact of ghost fishing on this fishery. This study offers the most accurate estimates currently available for the Barents Sea fishery. These findings underscore the importance of pragmatic approaches in addressing ghost fishing and emphasize the need for future studies to examine factors such as the presence of live crabs in lost pots. The results of this study also emphasize the need to adopt measures to reduce gear loss and mitigate the impacts of ghost fishing on this fishery.

## Data Availability

The datasets generated during and analysed in this study are available from the following repository: DOI: 10.6084/m9.figshare.28752449.
